# Effective Cytotoxic T Lymphocyte Targeting of Persistent HIV-1 during Antiretroviral Therapy Requires Priming of Naive CD8^+^ T Cells

**DOI:** 10.1128/mBio.00473-16

**Published:** 2016-05-31

**Authors:** Kellie N. Smith, Robbie B. Mailliard, Paolo A. Piazza, Will Fischer, Bette T. Korber, Ronald J. Fecek, Deena Ratner, Phalguni Gupta, James I. Mullins, Charles R. Rinaldo

**Affiliations:** a Department of Microbiology and Molecular Genetics, University of Pittsburgh, Pittsburgh, Pennsylvania, USA; b Department of Infectious Diseases and Microbiology, University of Pittsburgh, Pittsburgh, Pennsylvania, USA; c Theoretical Biology and Biophysics Group, Los Alamos National Laboratory, Los Alamos, New Mexico, USA; d Department of Microbiology, University of Washington, Seattle, Washington, USA; e Department of Pathology, University of Pittsburgh School of Medicine, Pittsburgh, Pennsylvania, USA

## Abstract

Curing HIV-1 infection will require elimination of persistent cellular reservoirs that harbor latent virus in the face of combination antiretroviral therapy (cART). Proposed immunotherapeutic strategies to cure HIV-1 infection include enhancing lysis of these infected cells by cytotoxic T lymphocytes (CTL). A major challenge in this strategy is overcoming viral immune escape variants that have evaded host immune control. Here we report that naive CD8^+^ T cells from chronic HIV-1-infected participants on long-term cART can be primed by dendritic cells (DC). These DC must be mature, produce high levels of interleukin 12p70 (IL-12p70), be responsive to CD40 ligand (CD40L), and be loaded with inactivated, autologous HIV-1. These DC-primed CD8^+^ T cell responders produced high levels of gamma interferon (IFN-γ) in response to a broad range of both conserved and variable regions of Gag and effectively killed CD4^+^ T cell targets that were either infected with the autologous latent reservoir-associated virus or loaded with autologous Gag peptides. In contrast, HIV-1-specific memory CD8^+^ T cells stimulated with autologous HIV-1-loaded DC produced IFN-γ in response to a narrow range of conserved and variable Gag peptides compared to the primed T cells and most notably, displayed significantly lower cytolytic function. Our findings highlight the need to selectively induce new HIV-1-specific CTL from naive precursors while avoiding activation of existing, dysfunctional memory T cells in potential curative immunotherapeutic strategies for HIV-1 infection.

## IMPORTANCE

Current immunotherapeutic approaches aim to enhance antiviral immunity against the HIV-1 reservoir; however, it has yet to be shown whether T cells from persons on cART can recognize and eliminate virus-infected cells. We show that in persons on cART a personalized medicine approach using their dendritic cells to stimulate their naive T cells induces potent effector CTL *in vitro* that recognize and eradicate HIV-1-infected CD4^+^ T cells. Additionally, we show that the same stimulation of existing memory T cells results in cytokine secretion but limited effector function. Our study demonstrates that the naive T cell repertoire can recognize persistent HIV-1 during cART and supports immunotherapy strategies for an HIV-1 cure that targets naive T cells, rather than existing, dysfunctional, memory T cells.

## INTRODUCTION

Combination antiretroviral therapy (cART) has greatly reduced the morbidity and mortality associated with chronic HIV-1 infection. Nevertheless, a stable latent viral reservoir persists in blood and gut-associated lymphoid tissues and other lymphatics even after long-term virus-suppressive therapy (1–4), presenting a major barrier to viral control and eradication. Importantly, anti-HIV-1 CD4^+^ and CD8^+^ T cell responses decrease during cART, presumably due to weak antigenic stimulation consequent to lower viral load (5–7). Therefore, while partial immune reconstitution is achieved during cART, the antiviral functionality of the reconstituted immune system is limited (8).

A personalized medicine approach, based on induction of a broad and robust cytotoxic T lymphocyte (CTL) response specific for the patient’s own unique, autologous virus, has previously been proposed for eliminating HIV-1-infected cells (9–12). Unfortunately, escape mutations are prevalent during the early and chronic phases of HIV infection (13, 14), and the virus evades host CTL responses through chronic immune activation and dysregulation (15, 16). Therapeutic approaches have therefore aimed to enhance anti-HIV-1 CTL activity in persons on cART, when the viral burden and mutation rate are minimized and partial immune reconstitution has occurred (13). However, most latently infected cells do not express viral proteins during virus-suppressive cART and are therefore undetectable by the immune system (17).

To control HIV-1 replication and ultimately cure infection, a “shock and kill” approach has been proposed. In this concept, cells harboring the latent HIV-1 reservoir are induced to produce viral protein antigens (“shock”), coincident with a potent immunotherapy that induces CTL specific for the patient’s autologous virus (“kill”) (18). Such immunotherapies aim to reactivate HIV-1-specific memory CD8^+^ T cells in persons on cART. Our previous studies support the potential of dendritic cells (DC) to induce strong, antigenically broad, high-magnitude HIV-1-specific memory CD8^+^ T cell responses during chronic HIV-1 infection (untreated and treated) (12, 19–23). However, we have also shown that, although HIV-1-specific memory CTL maintain long-term cytolytic function against their cognate antigens, they selectively produce inflammatory factors in the absence of cytolysis upon secondary encounter with epitope variants (24). This has obvious implications in a DC-based immunotherapy, wherein ineffective or dysfunctional memory recall CTL responses can be maintained throughout infection and could be selectively induced against surviving HIV-1 variants.

One alternative to reactivating memory CD8^+^ T cells is stimulating naive CD8^+^ T cells during immunotherapy to generate new primary CTL that specifically target the surviving autologous HIV-1 reservoir. Many factors are involved in generating CTL from naive CD8^+^ T cells, including the number and phenotypic repertoire of the naive T cell precursors, helper CD4^+^ T cell contributions, and the nature of the antigen-presenting cell/naive T cell interaction (25). Indeed, we have previously shown that mature, antigen-loaded DC producing high levels of interleukin 12p70 (IL-12p70) can induce primary HIV-1-specific CD8^+^ T cells in HIV-1-naive donors (24, 26). It is currently unclear, however, whether the repertoire and function of naive T cells in HIV-1-infected persons on cART are sufficient to enable a response to such a therapy. Moreover, abnormalities in T cell receptor (TCR) diversity and naive T cell function, including responsiveness to neo-antigens, occur during chronic HIV-1 infection (27). Technical limitations have impeded these types of analyses with HIV-1, as evaluating the induction of primary responses to autologous HIV-1 antigen in persons on cART requires the following: (i) mature DC capable of producing IL-12p70 that will maintain production following interaction with CD4^+^ T cells in the lymph node, (ii) isolation of autologous HIV-1 from latently infected CD4^+^ T cells as an antigenic source representative of the persistent virus reservoir, and (iii) a highly pure population of naive CD8^+^ T cells, as the presence of contaminating memory T cells could compete with and mask primary T cell responses.

We hypothesized that naive CD8^+^ T cells from persons on cART can be successfully primed by properly programmed DC to respond to the autologous HIV-1 more effectively than the pool of preexisting memory CD8^+^ T cells. To address this hypothesis, we established a novel, clinically relevant *in vitro* model of DC immunotherapy to evaluate T cell antigen specificity and antiviral capacity against autologous HIV-1 following DC stimulation of naive and memory T cells. For the first time, we show that naive CD8^+^ T cells from chronic HIV-1-infected persons on cART can be primed to specifically target conserved and variable regions of Gag and kill CD4^+^ T cells infected with autologous HIV-1 *in vitro*. In contrast, memory CD8^+^ T cells exposed to the same stimulation exhibited limited reactivity as determined by cytokine production, and were substantially less effective at killing CD4^+^ T cells infected with autologous HIV-1. Our findings support the selective priming of naive CD8^+^ T cells in HIV-1-infected persons on cART using a DC-based therapeutic strategy to target the autologous HIV-1 reservoir.

## RESULTS

### A novel *in vitro* model of DC immunotherapy targeting naive and memory T cell effector responses against the autologous HIV-1 in participants on cART.

Virus eradication strategies have proposed that potent CTL targeting of the autologous HIV-1 reservoir could be generated using DC-based immunotherapeutic approaches (28). The character and quality of a DC-induced T cell response greatly depend on the mode of DC activation (29). Optimal DC-induced T cell responses require DC with a mature status (enhanced surface expression of T cell costimulatory molecules) and lymphoid homing potential (expressing chemokine [C-C motif] receptor 7 [CCR7]) and capable of producing IL-12p70 upon interaction with T cells (30). Importantly, activation of DC by CD40L-expressing CD4^+^ T cells allows DC to act as effective mediators of T helper (Th) signals for the induction of long-lived CTL-based immunity (31). Unfortunately, Th cells are targeted and impaired as a result of HIV-1 infection. Therefore, we chose to explore the use of recombinant human CD49 ligand (rhCD40L) as both a DC maturation factor and surrogate for CD4^+^ T cell “help” in our *in vitro* HIV-1-specific cytotoxic T cell priming studies.

**FIG 1:**
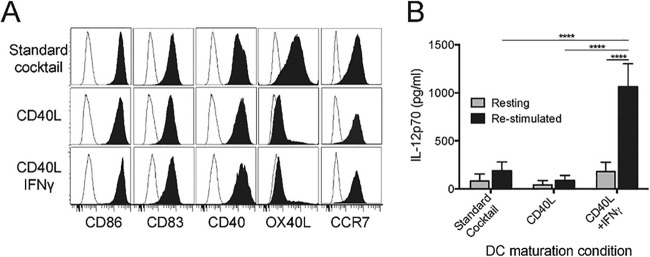
Phenotypic and functional characterization of differentially matured DC. Immature DC were stimulated for 48 h with a cytokine cocktail (32), CD40L alone, or a combination of CD40L and IFN-γ. (A) Maturation status was confirmed by flow cytometric staining for the T cell costimulatory molecules CD86, CD83, and CD40, the Th2-promoting molecule OX40L, and the lymphoid homing receptor CCR7. (B) Mature DC were evaluated for their polarization status by measuring IL-12p70 content in supernatants following secondary stimulation with (re-stimulated) or without (resting) CD40L. Values that are significantly different (*P* < 0.0001) are indicated by a bar and four asterisks.

Monocyte-derived DC from healthy HIV-1-negative individuals were generated and differentially matured with either a maturation cytokine cocktail (32) consisting of tumor necrosis factor alpha (TNF-α), IL-1β, IL-6, and prostaglandin E2 (PGE2) that was used in the HIV-1 therapeutic vaccine trial reported by Garcia et al. (33), or CD40L with and without IFN-γ (20–22, 34). All three stimulation conditions produced DC with a mature phenotype, showing expression of the typical maturation markers CD86 and CD83, the lymphoid homing receptor CCR7, and the CD40L receptor CD40 (Fig. 1A). The OX40 ligand (OX40L), which promotes Th2 differentiation (35, 36), was highly expressed only on DC matured using the standard cocktail.

The ability of differentially matured DC to maintain their IL-12p70-producing capacity was assessed by harvesting and replating the mature DC in fresh media, and collecting 24-h supernatants from DC cultured either in their resting state or following restimulation with the Th cell-associated activation signal CD40L. In the resting state, all DC produced similarly low quantities of IL-12p70. However, DC matured using the combination of CD40L and IFN-γ were most responsive when stimulated with CD40L, producing a significantly enhanced amount of IL-12p70 compared to the other DC maturation conditions (*P* < 0.0001; Fig. 1B). This suggests, in accord with our previous study (34), that these DC would maintain responsiveness to Th cells *in vivo* if therapeutically administered. As a result, we elected to use this maturation approach for our DC-based *in vitro* immunotherapy studies.

To target the residual viral reservoir with DC immunotherapy during cART initiated in chronic HIV-1 infection, it is unclear whether new, highly effective CTL need to be generated from naive precursors or whether endogenous memory CTL can be stimulated to overcome their dysfunctional status. Furthermore, it is not known whether the primed naive CD8^+^ T cell repertoire is diverse enough to recognize the conserved and variable regions of HIV-1 proteins expressed in the reservoir, as a key hypothesis of CTL failure in chronic infection is that the virus mutates to evade host recognition. We therefore developed a novel approach to evaluate the capacity of naive and memory T cells to respond to primary and secondary DC stimulation, respectively, in chronic HIV-1-infected individuals on virus-suppressive cART.

Leukapheresis was performed on HIV-1-infected participants of the Multicenter AIDS Cohort Study (MACS) (37, 38) who had documented time of HIV-1 seroconversion and progressive, untreated HIV-1 infection for >6 years prior to virus suppressive cART for >10 years (see Fig. S1 in the supplemental material). We have previously assessed these participants for their longitudinal memory CD8^+^ T cell cytokine responses to autologous Gag and Env epitopes (12). Leukapheresis products were used to generate monocyte-derived DC, to isolate autologous HIV-1 from CD4^+^ T cells, and to obtain T cell subsets utilized in the current study. Naive and memory CD4^+^ and CD8^+^ T cells were purified using fluorescence-activated cell sorting (FACS). The gating strategy for isolating naive CD4^+^ T cells (CD3^+^, CD4^+^, CD62L^+^, CD45RA^+^, CCR7^+^, and CD31^+^) and CD8^+^ T cells (CD3^+^, CD8^+^, CD62L^+^, CD45RA^+^, and CCR7^+^) is shown in Fig. S2A. Memory CD8^+^ T cells were isolated by gating on CD4^+^ and CD8^+^ T cells that did not fall within the “naive” T cell gate. Additional studies accounted for stem cell memory T (Tscm) CD8^+^ and CD4^+^ T cells being within the naive T cell populations by removing these using the Tscm marker CD95 to result in naive CD4^+^ (CD3^+^, CD4^+^, CD45RA^+^, CD27^+^, CCR7^+^, and CD95^−^) and naive CD8^+^ (CD3^+^, CD8^+^, CD45RA^+^, CD27^+^, CCR7^+^, and CD95^−^) T cells (Fig. S2C).

**FIG 2:**
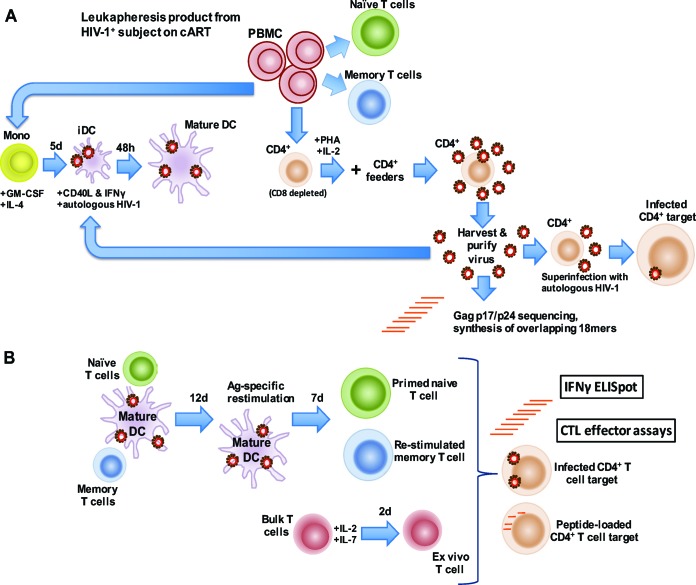
Stimulating naive and memory CD8^+^ T cells with autologous HIV-1. An *in vitro* model of DC immunotherapy was developed to assess the potential of naive and memory CD8^+^ T cells to respond to stimulation against autologous HIV-1. (A) Reactivated HIV-1 was isolated from CD8^+^ T cell-depleted, activated CD4^+^ T cells (CD8 depleted) obtained during cART and cultured with mitogen-activated, autologous CD4^+^ T cells (CD4^+^ feeders). Immature DC (iDC) were loaded with purified, autologous, AT-2-inactivated HIV-1, and matured using CD40L and IFN-γ. Gag p17 and p24 were sequenced from purified virus, and a peptide library was generated representing the consensus HIV-1 Gag p17 and p24 sequences from each participant. Abbreviations: Mono, monocytes; GM-CSF, granulocyte-macrophage colony-stimulating factor; 5d, 5 days; (B) Mature, antigen-loaded DC (mature DC) were used to stimulate naive or memory CD4^+^ and CD8^+^ T cells in an *in vitro* model of DC immunotherapy. Following a 19-day stimulation protocol, CD8^+^ T cells were evaluated for CTL effector function by IFN-γ ELISpot and an HIV-1-infected, autologous CD4^+^ T cell elimination assay as well as a CTL assay against autologous CD4^+^ T cell targets loaded with autologous HIV-1 Gag peptides. Ag, antigen.

We verified the purity of the naive T cell populations by confirming the ablation of memory CD8^+^ T cell responses to a potent pool of major histocompatibility complex (MHC) class I-restricted, cytomegalovirus, Epstein-Barr virus, and influenza A virus peptide epitopes (39), and to a pool of autologous Gag p17 and p24 peptides as determined by IFN-γ enzyme-linked immunosorbent spot assay (ELISpot) (see Fig. S2B in the supplemental material) as well as CD107a expression by flow cytometry (Fig. S2D). Autologous, reactivated HIV-1 was purified, and sequencing was performed on *gag* p17 and p24 for each participant (12). We inactivated this purified, autologous virus with aldrithiol-2 (AT-2) (40) and loaded the inactivated virus preparation into autologous DC for use in afferent T cell stimulation. Active autologous HIV-1 was used to superinfect preactivated, autologous CD4^+^ T cells for use as targets in CTL assays. The workflow for isolating the virus and T cell populations, and generating HIV-1-loaded DC and superinfected CD4^+^ T cell targets, is shown in Fig. 2A.

We used the CD40L and IFN-γ-matured, autologous, HIV-1-loaded DC to stimulate naive and memory CD4^+^ and CD8^+^ T cells as an *in vitro* model of DC immunotherapy. Although we are ultimately evaluating CD8^+^ CTL function, naive CD4^+^ T cells were included in the priming to serve a “helper” role during CTL induction (26). The workflow for generating HIV-1 antigen-specific, primed CD8^+^ T cells from naive precursors, and stimulated memory CD8^+^ T cells from memory precursors, is shown in Fig. 2B. At the end of the 19-day stimulation protocol, these primed and stimulated memory T cells were tested in endpoint immunological assays.

### DC-primed naive CD8^+^ T cells respond to autologous HIV-1 antigen challenge with enhanced breadth and magnitude compared to *ex vivo* and stimulated memory CD8^+^ T cells.

**FIG 3:**
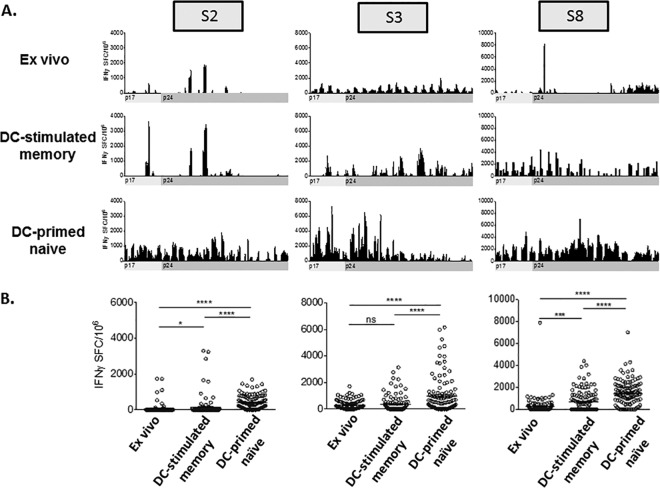
IFN-γ production by *ex vivo*, DC-stimulated memory, and DC-primed naive CD8^+^ T cells in response to 18-mers representing the autologous cART Gag p17 and p24 sequence. *Ex vivo*, DC-stimulated memory, and DC-primed naive CD8^+^ T cells from participants S2, S3, and S8 were stimulated with immature DC loaded with 18-mers representing the autologous Gag p17 and p24 consensus cART sequence. CD8^+^ T cell reactivity was evaluated by IFN-γ ELISpot. (A) The mean number of IFN-γ-producing spot-forming cells (IFNγ SFC) per 10^6^ responders observed in duplicate wells is shown for each peptide, with the mean background plus 2 standard deviations subtracted, except for the DC-stimulated memory responses for participant S8, in which peptide stimulations were performed once. (B) IFN-γ ELISpot responses to autologous HIV-1 Gag p17 and p24 18-mers were averaged for participants S2 (left), S3 (center), and S8 (right). Values that are significantly different are indicated by a bar and asterisks as follows: *, *P* < 0.05; ***, *P* < 0.001; ****, *P* < 0.0001. Values that are not significantly different (ns) are also indicated.

The inflammatory immune response of DC-stimulated memory CD8^+^ T cells and DC-primed naive CD8^+^ T cells to autologous HIV-1 antigen was evaluated *ex vivo*, after 2 days of culture, by IFN-γ ELISpot using overlapping 18-mer peptides representing the autologous Gag p17 and p24 consensus sequence of reactivated, autologous HIV-1 (Fig. 3). We first examined the antigenic breadth of CD8^+^ T cell responses to the autologous Gag peptide libraries. We detected *ex vivo* CD8^+^ T cell responses to 16/85 (18.8%) peptides in participant S2 that were comparable to memory responses to 19/85 (22.4%), and less than primed naive T cell responses to 77/85 (90.6%) peptides (Fig. 3A). We detected *ex vivo* CD8^+^ T cell responses to 64/84 (76.2%) peptides in participant S3 compared to 40/84 (47.6%) and 78/84 (92.9%) in the stimulated memory and primed naive T cells, respectively (Fig. 3A). Last, we observed *ex vivo* T cell responses to 23/84 (27.4%) peptides in participant S8 compared to 45/84 (53.6%) and 74/84 (88.1%) in stimulated memory and primed naive T cells, respectively (Fig. 3A). These data demonstrate variable recognition of autologous Gag antigens by *ex vivo* and DC-stimulated memory CD8^+^ T cells, with a consistently higher antigenic breadth of recognition by T cells primed from naive precursors.

We next determined whether the enhanced breadth seen in primed T cells was accompanied by an increase in magnitude. IFN-γ ELISpot responses to Gag p17 and p24 peptides were averaged for each participant and compared among the three T cell conditions (Fig. 3B). For each participant tested, we consistently noted higher mean IFN-γ production by primed naive T cells compared to *ex vivo* T cells and DC-stimulated memory T cells. Together, these data show that, while there is recognition of autologous Gag antigens by memory CD8^+^ T cells evaluated after short-term (*ex vivo*) or long-term stimulation, the antigenic breadth and magnitude are consistently greater when CD8^+^ T cells are primed from naive precursors.

### DC-primed naive CD8^+^ T cells respond with greater magnitude to autologous Gag peptide epitopes compared to DC-stimulated memory CD8^+^ T cells.

Targeting of MHC class I-restricted epitopes has been associated with CTL effector function and reductions in HIV-1 load (41–43). We therefore evaluated IFN-γ production to DC-stimulated memory and primed naive CD8^+^ T cell to the 18-mers containing known HIV-1 Gag p17 and p24, MHC class I-restricted epitopes as listed in the Los Alamos HIV Molecular Immunology Database. These 18-mers, the encompassed MHC class I epitopes and the cognate ELISpot responses detected by both T cell conditions, with affiliated statistical analyses, are shown for each participant in Tables 1 to 3. In participant S2, 25 18-mers contained known class I-restricted epitopes (Table 1). Of these 25, 11 induced significantly greater IFN-γ production in primed naive cells than DC-stimulated memory cells did, while only 6 peptides induced a greater response in the stimulated memory cells. Results from participant S3 were similar: 16 18-mers contained known epitopes, 5 of which induced a significantly greater response in primed T cells and none of which induced a greater response in the DC-stimulated memory cells (Table 2). In participant S8, 20 peptides contained known epitopes for this participant’s HLA alleles (Table 3). Because stimulation of memory cells in this participant consistently yielded enough only cells to perform singlet wells in the ELISpot assay, we could not calculate *P* values. We therefore determined a Δ value for each 18-mer and defined this as the difference in the DC-stimulated memory response compared to the primary response. A positive Δ value indicates a higher primary response, and a negative Δ value indicates a higher memory response. We then identified 95% confidence intervals for the positive and negative values separately; 18-mers with a Δ value falling outside this confidence interval were considered a substantial difference between the two cell types. In total, 6 18-mers induced Δ values outside the “positive” confidence interval, and 2 18-mers induced Δ values outside the negative confidence interval (Table 3).

**TABLE 1:**
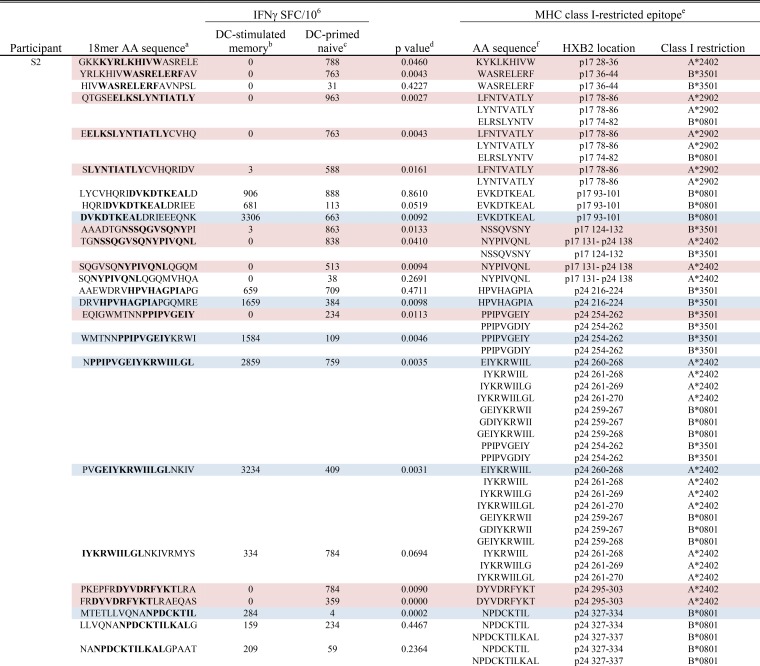
DC-primed naive and DC-stimulated memory CD8^+^ T cell responses detected in participant S2 to 18-mers containing autologous variants of known MHC class I-restricted epitopes

^a^ The amino acid (AA) sequences of 18-mers containing autologous variants of known MHC class I-restricted epitopes. The sequences spanning the known epitopes are shown in boldface type.

^b^ The mean IFN-γ response detected in DC-stimulated memory T cells is shown as the number of IFN-γ-producing spot-forming cells per 10^6^ responders (IFNγ SFC/10^6^).

^c^ The mean IFN-γ response detected in DC-primed naive T cells is shown as the number of IFN-γ-producing spot-forming cells per 10^6^ responders (IFNγ SFC/10^6^).

^d^ The *P* value as determined by a paired Student’s *t* test comparing mean IFN-γ production between DC-stimulated memory T cells and DC-primed naive T cells. An 18-mer that induced a significantly higher IFN-γ response in DC-primed naive cells is indicated by pink highlighting, whereas an 18-mer that induced a significantly higher response in DC-stimulated memory cells is indicated by blue highlighting.

^e^ Known MHC class I-restricted epitopes for each subject’s HLA alleles as determined using the Los Alamos Database. For the HXB2 location, the protein and the coordinates are shown.

^f^ Amino acid sequence of known MHC class I-restricted HIV-1 Gag epitopes.

**TABLE 2:**
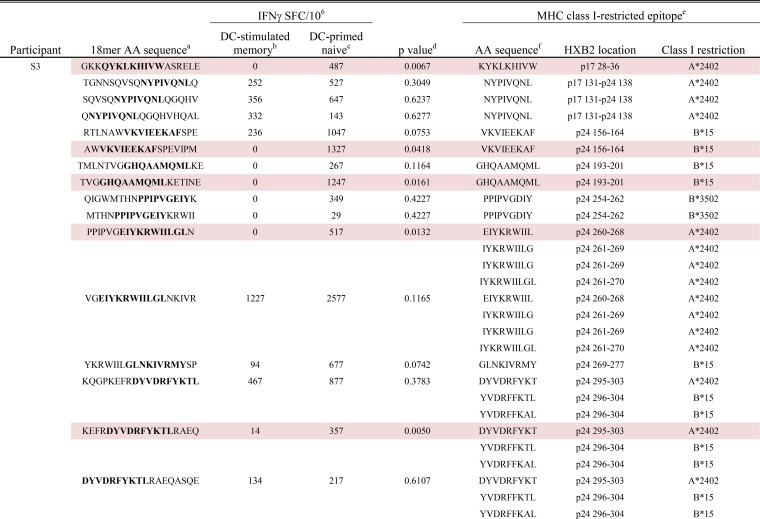
DC-primed naive and DC-stimulated memory CD8^+^ T cell responses detected in participant S3 to 18-mers containing autologous variants of known MHC class I-restricted epitopes^g^

g For footnotes *a* to *f*, see the footnotes to Table 1.

**TABLE 3:**
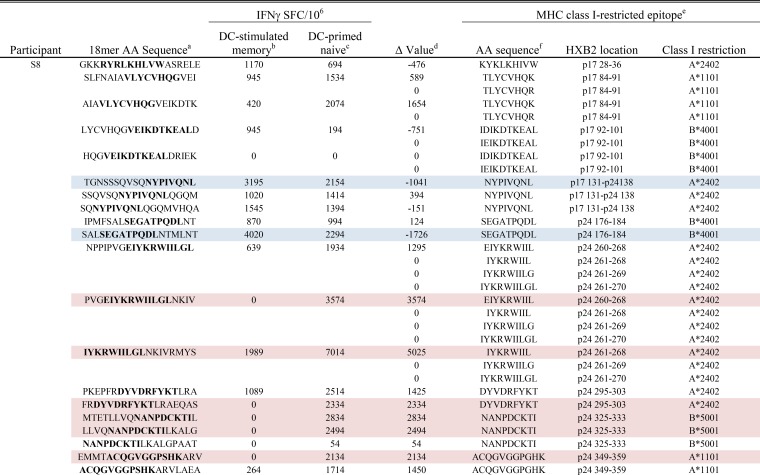
DC-primed naive and DC-stimulated memory CD8^+^ T cell responses detected in participant S8 to 18-mers containing autologous variants of known MHC class I-restricted epitopes

^a^ The amino acid (AA) sequences of 18-mers containing autologous variants of known MHC class I-restricted epitopes. The sequences spanning the known epitopes are shown in boldface type.

^b^ The mean IFN-γ response detected in DC-stimulated memory T cells is shown as the number of IFN-γ-producing spot-forming cells per 10^6^ responders (IFNγ SFC/10^6^).

^c^ The mean IFN-γ response detected in DC-primed naive T cells is shown as the number of IFN-γ-producing spot-forming cells per 106 responders (IFNγ SFC/10^6^).

^d^ The  Δ value was determined by subtracting the mean IFN-γ response in DC-stimulated memory T cells from the response detected in DC-primed naive T cells to all 84 18-mers that were evaluated in this subject. A 95% confidence interval was calculated for all positive (DC-primed naive T cell response > DC-stimulated memory T cell response) Δ values. An 18-mer that had a Δ value that fell outside the positive 95% confidence interval is indicated by pink highlighting, whereas an 18-mer with a Δ value that fell outside the negative 95% confidence interval is indicated by blue highlighting.

^e^ Known MHC class I-restricted epitopes for each subject’s HLA alleles as determined using the Los Alamos Database. For the HXB2 location, the protein and the coordinates are shown.

^f^ Amino acid sequences of known MHC class I-restricted HIV-1 Gag epitopes.

Thus, we found a consistent propensity for epitope-containing 18-mers to induce greater IFN-γ responses in primed naive rather than DC-stimulated memory CD8^+^ T cells. Of note, in each participant, there was a significantly greater primary response to an 18-mer containing the known HLA-A*2402-restricted p24 DYVDRFYKT epitope. In two out of three participants, we detected a greater primary response specific for 18-mers containing the immunodominant HLA-A*2402-restricted p17 KYKLKHIVW and p24 EIYKRWIIL epitopes.

Notably, these data show that *de novo* CD8^+^ T cell IFN-γ responses that target known CTL epitopes can be generated against autologous HIV-1 by priming naive T cells from persons on long-term suppressive cART. The primed responses were greater than the memory responses. This supports the idea that *de novo* activation of naive CD8^+^ T cell precursors by DC results in recognition of a broader range of autologous HIV-1 antigens, responding with higher magnitude, than those generated by stimulating endogenous memory T cells with DC.

Important interplay between protein conservation in HIV-1 and immune targeting has been postulated (44), including the possibility that immunodominance benefits viral persistence (45) by preferentially exposing mutable epitopes. It has additionally been suggested that targeting conserved, lower-entropy regions of *gag* could be associated with a clinical benefit (46), particularly when targeting the latent HIV-1 reservoir (9). The primary epitope targeting described here raised the intriguing possibility that the new set of epitopes would be qualitatively different in terms of their conservation. That is, the primary T cell responses could have side-stepped the diversion of the immune response by HIV-1 toward high-entropy epitopes and preferentially targeted more-beneficial, low-entropy epitopes that have higher fitness costs for escape.

**FIG 4:**
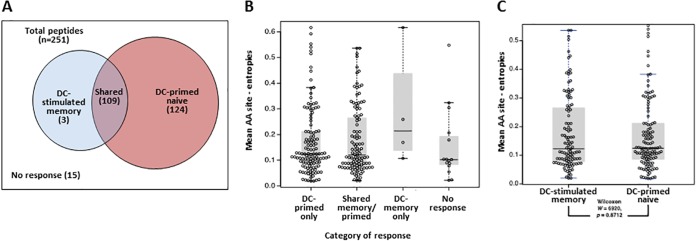
Shannon entropy scores of 18-mers targeted by DC-stimulated memory and DC-primed naive T cells. 18-mers targeted by DC-primed naive and DC-stimulated memory T cells were evaluated for differences in entropy. (A) Categorization of the 251 peptide/subject combinations (3 participants, 208 unique peptides) in terms of positive IFN-γ ELISpot response and cell population (DC-stimulated memory cells or DC-primed naive cells). (B) Mean amino acid (AA) site entropy scores of all 18-mer peptides tested, broken down by response category as shown in panel A. Each symbol represents the value for a peptide/subject combination, the short horizontal line represents the mean value for the response category, and the shaded boxes represent the 25th and 75th quartiles. (C) Mean amino acid site entropy scores of 18-mer peptides for peptides involved in either DC-stimulated memory responses (includes “shared” peptides evoking both memory and primary responses) or DC-primed naive cell only responses. Significance was tested using a two-sided Wilcoxon test (*P* = 0.8712).

We used Shannon entropy to test for differences in conservation between two groups of epitopes: those that were newly targeted following priming of naive T cells by DC and those targeted by memory T cells that were stimulated by DC. Assessment of the autologous 18-mer Gag peptides tested by IFN-γ ELISpot for each of the three participants showed that, of 251 patient/peptide combinations, 236 evoked above-background responses. These were three 18-mers that were positive only in DC-stimulated memory cells, 109 that were positive in both the DC-stimulated memory and priming conditions, and 124 that were only seen in the primed naive T cells (Fig. 4A). There were 15 18-mers to which no response was detected in either T cell condition. We then compared peptide conservation by calculating the Shannon entropy score for the 18-mers found in each response category (DC-primed naive only, DC-stimulated memory only, shared, and no response). There were no significant differences in entropy between 18-mers that were targeted by only DC-primed naive T cells, DC-stimulated memory T cells, both populations, and none of the populations (Fig. 4B). Additionally, when comparing the entropy of reactive 18-mers, there were no differences observed between DC-stimulated memory and DC-primed naive T cells (two-sided Wilcoxon test, *P* = 0.87; Fig. 4C).

**FIG 5:**
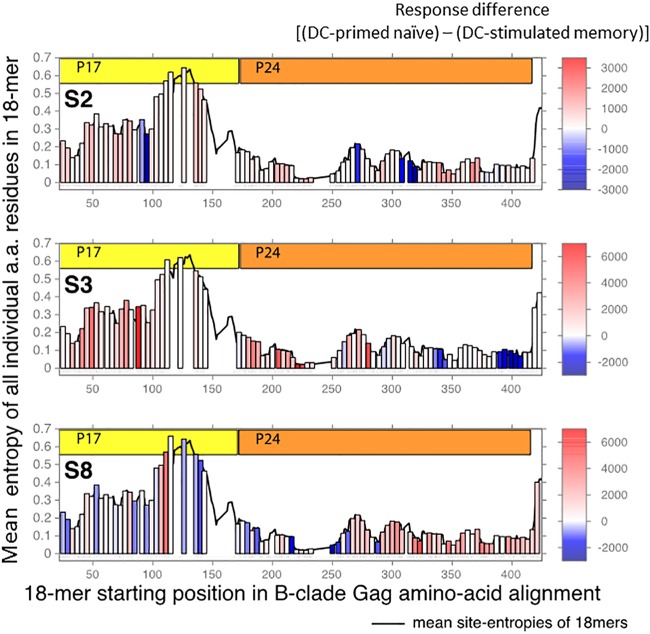
Peptide entropy is not predictive of higher-magnitude T cell responses. For each 18-mer peptide tested, the mean site entropy was calculated for each column in the LANL HIV Sequence Database 2014 Gag protein “filtered web alignment” (see text). The entropy of each tested peptide is shown as a vertical bar, positioned left to right by its position in the alignment and colored by the arithmetic difference between the DC-primed naive and DC-stimulated memory T cell responses as seen in Tables 1 to 3 (red shows higher-magnitude DC-primed naive T cell response, and blue shows higher-magnitude DC-stimulated memory T cell response; scale bars at right). The mean site entropies for all 18-mers in the alignment are shown as a black line. a.a., amino acid.

We then tested the relationship between IFN-γ response magnitude (Fig. 3; Tables 1 to 3) and peptide entropy for peptides targeted by DC-primed naive and DC-stimulated memory T cells using a Kendall tau test (47). We observed no preferential targeting of lower-entropy 18-mers by either of the T cell subsets and no significant relationship between magnitude of response and peptide entropy in any of the study participants (Fig. 5).

### Primed naive CD8^+^ T cell eradication of autologous HIV-1-infected CD4^+^ T cells.

**FIG 6:**
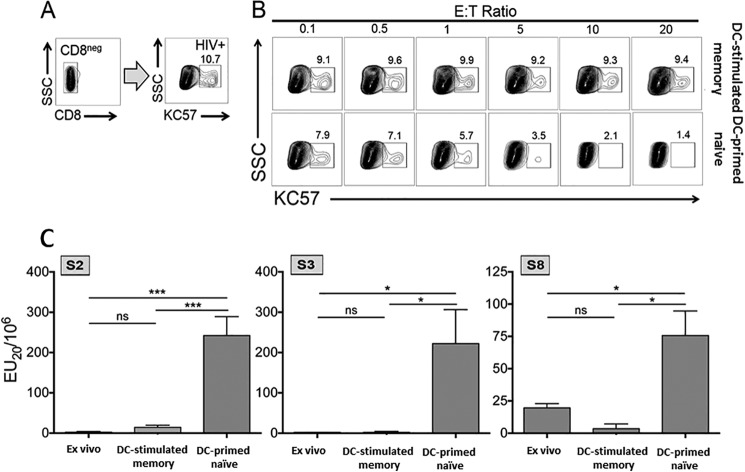
Primed naive CTL eliminate CD4^+^ T cells infected with autologous HIV-1. *Ex vivo*, DC-stimulated memory, and primed naive CD8^+^ T cells were evaluated for cytolytic function against the autologous HIV-1. (A) Autologous CD4^+^ T cells were superinfected with autologous HIV-1 derived from the same time point, with 10 to 30% of the cells staining positive for the HIV-1 core antigen KC57. SSC, side scatter. (B) CD8^+^ T cells were cocultured with autologous virus-infected CD4^+^ T cells at various E:T ratios for 18 h. Flow cytometry was used to determine the percentage of CD4^+^ T cells that were positive for HIV-1 p24 following the coculture. (C) To quantify CTL, we applied the formula for calculating lytic units to our flow cytometry-based assay. Mean effector units (EU_20_) plus standard deviations of three experiments for *ex vivo*, DC-stimulated memory, and DC-primed naive CD8^+^ T cells are shown for participants S2, S3, and S8. Statistical significance or the lack thereof is indicated as follows: *, *P* < 0.05; ***, *P* < 0.001; ns, not significant.

We next determined whether the enhanced breadth and magnitude of primed CD8^+^ T cells were associated with increased CTL targeting of CD4^+^ T cells infected with autologous HIV-1. We used a modified version of a previously developed coculture system (48) to assess CTL elimination of HIV-1-infected CD4^+^ T cells. Briefly, CD4^+^ T cells from each participant were infected *in vitro* with autologous HIV-1 to generate a target population with 10 to 30% of the cells staining positive for the HIV-1 Gag p24 core antigen. Representative HIV-1-infected targets generated for participant S3 are shown in Fig. 6A. Purified CD8^+^ T cells from the DC-stimulated memory and DC-primed naive T cell conditions were cocultured with autologous, HIV-1-infected CD4^+^ T cells at various effector-to-target cell (E:T) ratios for 18 h. The cell cultures were stained for T cell markers and p24 core antigen to evaluate elimination of autologous HIV-1-infected CD4^+^ T cells. A representative experiment for participant S3 is shown in Fig. 6B. In this participant, primed naive T cells demonstrated a consistent dose response in antiviral activity, reducing HIV-1-infected CD4^+^ T cells by 87% at the highest, 20:1 E:T ratio. In contrast, DC-stimulated memory T cells did not display a dose response in reduction of virally infected cells, reducing the percentage of infected CD4^+^ T cells by approximately 13% at each E:T ratio. Similar results were found with CD8^+^ memory and primed T cells from the other two participants and in additional repetitive experiments with participant S3 (data not shown). To more quantitatively measure lytic activity, we calculated the effector units (EU_20_) for each T cell condition by applying the lytic unit formula (49), typically used in classical chromium release cytotoxicity assays, to our flow cytometry-based method. Primed naive T cell effector function was significantly greater than the *ex vivo* and DC-stimulated memory T cell condition in all three participants (Fig. 6C). Moreover, there was no difference between *ex vivo* and DC-stimulated memory T cell reactivity.

**FIG 7:**
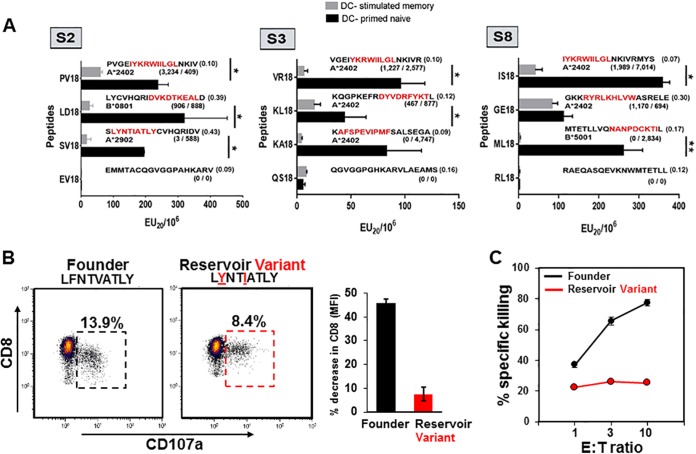
DC-primed naive compared to DC-stimulated memory CD8^+^ T cells show higher CTL activity against targets loaded with select 18-mers from the autologous cART Gag p17/p24 sequences. (A) DC-primed naive and DC-stimulated memory CD8^+^ T cells were assessed by flow cytometry for cytolytic activity against autologous CD4^+^ T cells loaded with peptides found to be associated with a range of magnitudes of IFN-γ ELISpot responses (see Tables 1 to 3). The numbers of spots are reported within the parentheses below each peptide sequence for DC-stimulated memory T cells (before the slash) and DC-primed naive T cells (after the slash). The bottom data set within the vertical panels (peptides EV18, QS18, RL18) shows CTL activity against peptides that were not associated with either DC-stimulated memory or DC-primed naive IFN-γ production. For each peptide, the relevant epitope sequence is shown in red, and the HLA-restricting allele is shown below each sequence. Data are means plus standard errors of 3 or 4 replicates within the CTL assay of DC-stimulated and DC-primed naive CD8^+^ T cells. (B) (Left) Flow cytometry analysis of CD107a and CD8 surface expression levels of DC-stimulated memory CD8^+^ T cells from participant S2 following exposure to autologous HIV-1 founder epitope peptide (LFNTVATLY) and a reservoir-associated variant (LYNTIATLY [mutant amino acids underlined]). (Right) The bar graph represents relative decrease in CD8 expression (mean fluorescence intensity [MFI]) between antigen-responsive CD8^+^ T cells before and after antigen stimulation. (C) Cytotoxicity of autologous CD4^+^ T cell targets expressing either the HIV-1 founder epitope LFNTVATLY or reservoir variant epitope LYNTIATLY following 18-h incubation with S2 DC-stimulated memory CD8^+^ T cells.

We next directly assessed the autologous HIV-1 peptide-specific capacity of CTL killing by the DC-primed naive and DC-stimulated memory CD8^+^ T cells. For this, we developed a colorimetric, flow cytometry assay that measures the cytolysis of CD4^+^ T cells loaded with HIV-1 Gag 18-mer peptides. Through differential dye labeling of antigen-negative and antigen peptide-loaded CD4^+^ T cell targets using violet 450 (v450; violet) and carboxyfluorescein succinimidyl ester (CFSE; green), respectively, we were able to measure the selective loss of peptide antigen-loaded targets after their incubation with increasing numbers of effector CTL (see Fig. S4 in the supplemental material). For each participant, one set of three autologous 18-mer peptides was used in these CTL assays, each containing MHC class I-restricted 9- or 10-mer epitopes (8 known and 1 predicted) with a range in magnitude in their IFN-γ-inducing capacity in DC-primed naive and DC-stimulated memory T cells (Tables 1 to 3). A negative-control peptide was also included for each participant that did not contain a known Gag epitope. The results indicate that while lytic activity was detected using the DC-stimulated memory CD8^+^ T cells against different antigenic peptides, cytotoxic activity was consistently greater in the primed naive CD8^+^ T cells for all three peptides representing Gag epitopes in each study participant (Fig. 7A). Lytic activity was minimal or absent in response to the three control Gag peptides. Moreover, the overall, combined CTL reactivity to the nine Gag epitopes was 7-fold greater for the DC-primed naive versus the DC-stimulated memory T cells, i.e., mean (± standard error [SE]) EU_20_/10^6^ T cells of 197 (±24.5) compared to 28.1 (±5.8) (*P* < 0.0001), respectively. This is in contrast to the broad range of IFN-γ production that these same peptides induced in both the DC-primed naive and DC-stimulated memory CD8^+^ T cells (Tables 1 to 3).

Importantly, these data revealed that through DC priming of naive CD8^+^ T cells, CTL can be generated to effectively target widely expressed, highly conserved (low-entropy) antigenic targets, e.g., HLA-A*24.02-restricted epitope IYKRWIILGL shared by all three participants, as well as more-variable regions that include reservoir-associated CTL escape variants such as the HLA-A*29.02-restricted epitope LYNTIATLY in participant S2. We were able to determine that such epitopes were indeed CTL escape variants through longitudinal viral sequencing studies previously performed (12, 23) and by subsequently challenging the DC-stimulated memory CD8^+^ T cells with antigen derived from the founder HIV-1 sequences or the reservoir-associated HIV-1 sequences (Fig. 7B). In doing so, we found that while these memory CD8^+^ T cells failed to produce IFN-γ when exposed to the reservoir-associated variant antigen as determined by ELISpot (Fig. 7A) and flow cytometry (data not shown), they were in fact responsive to both founder- and reservoir-derived antigen variants as determined through antigen-induced expression of CD107a (Fig. 7B). However, this response was notably stronger toward the founder antigen and was accompanied by a strong downregulation of CD8 expression, a phenomenon associated with full effector function (24). Thus, when tested in our killing assay, CD4^+^ T cell targets loaded with the founder-associated antigen were eliminated, while targets presenting the reservoir-associated epitope variants were spared (Fig. 7C).

These results indicate that autologous DC can induce primary CTL from naive precursors and that these *de novo* CTL are superior to DC-stimulated memory CTL in targeting autologous HIV-1. The DC-stimulated memory CTL antiviral function was indistinguishable from that observed in *ex vivo* CD8^+^ T cells, showing that CTL effector function was not enhanced by either short- or long-term DC stimulation of memory T cells. Taken together, these findings demonstrate that naive CD8^+^ T cells in participants on cART can be primed by DC to recognize virus from reactivated autologous HIV-1, whereas memory CD8^+^ T cells exposed to this same stimulation have limited effector function and fail to effectively target these CD4^+^ T cells.

## DISCUSSION

The persistent HIV-1 reservoir that is maintained predominantly in CD4^+^ T cells in persons on cART is the major barrier to curing HIV-1 infection (50). Recent evidence suggests that HIV-1-specific CTL are essential for reducing these persistent viral reservoirs (9, 11). Barriers to this approach include the genetic diversity in the reservoir with a predominance of viral immune escape variants, and exhausted and dysfunctional T cells (51). To overcome these barriers, we propose that immunotherapies can be designed to maximize the afferent arm of DC priming of naive CD8^+^ T cells through selective programming of the DC pathway and presentation of the patient’s autologous viral reservoir, thereby maximizing the efferent arm of CTL-mediated killing. In support of this approach, we show here for the first time that autologous DC that are matured and programmed to produce large amounts of IL-12p70 effectively induced primary responses *in vitro* in naive CD8^+^ T cells against autologous HIV-1 antigen from participants on long-term, virus-suppressive cART. Prior to this study, it was unclear whether naive CD8^+^ T cells in these individuals were sufficiently restored in function and repertoire to respond to primary stimulation against the highly mutated HIV-1 reservoir. Indeed, we found that these naive CD8^+^ T cells can be primed to the participant’s persistent viral reservoir by DC loaded with autologous HIV-1 to produce high levels of IFN-γ to a broad array of autologous Gag antigens. Stimulation of memory CD8^+^ T cells with autologous virus-loaded DC also resulted in high levels of Gag peptide-specific IFN-γ production, in concordance with our previous results showing that DC can stimulate high-magnitude, antigenically broad, CD8^+^ T cell cytokine responses during cART (20, 22). IFN-γ responses were also observed in short-term, *ex vivo* cultures of T cells without DC, similar to previous studies showing recovery of such HIV-1-specific memory T cell reactivity during cART (7, 52, 53). This cytokine response, however, was of greater magnitude and breadth in the DC-stimulated memory T cells, and of the highest magnitude and breadth in the DC-primed naive T cells. Thus, CD8^+^ memory T cells in chronically infected individuals, even though in an exhausted state with high expression of coinhibitory molecules (51), can recover at least part of their ability to produce inflammatory cytokines such as IFN-γ while on cART.

A key finding in our study is that the DC-primed, HIV-1-specific, naive CD8^+^ T cells had robust antiviral cytotoxic activity. These primed CTL recognized and killed CD4^+^ T cells infected with autologous HIV-1 or loaded with a broad array of autologous Gag peptides. Remarkably, correspondent DC stimulation of memory CD8^+^ T cells resulted in much lower CTL lysis of either the autologous HIV-1-infected or Gag peptide-loaded CD4^+^ T cell targets. Thus, although DC stimulation of memory CD8^+^ T cells resulted in cytokine responsiveness to autologous HIV-1 antigen challenge, cytolytic targeting of CD4^+^ T cell targets remained comparatively inefficient. In this regard, few studies have compared CD8^+^ T cell cytokine production to cytolytic activity in persons on cART. Trabattoni et al. (54) reported that CD8^+^ T cells from persons on cART produced relatively high levels of cytokines including IFN-γ but expressed low levels of cytotoxic granules in response to HIV-1 Env peptides. Our findings are also in accord with the studies of Migueles and colleagues, highlighting the defective cytolytic function of cytokine-producing HIV-1-specific memory CD8^+^ T cells during chronic HIV-1 infection (55), a trait that persists even after viremia is controlled by cART (56). Deng et al. (9) recently described strong IFN-γ and CTL activity to heterologous HIV-1-infected, CD4^+^ T cell targets during cART mediated by autologous CD8^+^ memory T cells derived from peripheral blood mononuclear cells (PBMC) that were prestimulated for several days with consensus Gag peptides. Although we noted more-limited, anti-HIV-1 cytolytic activity by DC-stimulated memory CD8^+^ T cells, our model accounted for the impact of DC-mediated CD4^+^ T cell “help” in the priming, and long-term survival and functional capacity of CTL following secondary (restimulated on day 12) exposure to antigen (12), which occurred more than 7 days prior to the final cytotoxicity measurements. Thus, our system represents an *in vitro* surrogate for an effective therapeutic vaccine strategy for inducing robust CD8^+^ T cells responders with the capacity for functional expansion that is likely required to control autologous virus.

During untreated chronic HIV-1 infection, there is a predominance of highly mutated virus with numerous CTL escape mutations (57) that is stabilized but maintained during cART (58). Extensive analysis of our data showed that, using DC loaded with whole autologous HIV-1 in these long-term, chronically HIV-1-infected participants, IFN-γ production in DC-primed naive and DC-stimulated memory CD8^+^ T cells was induced by both low-entropy conserved and high-entropy variable regions of Gag. More importantly, DC-primed naive CD8^+^ T cells killed CD4^+^ T cells expressing both conserved and variable Gag epitopes much more efficiently than did DC-stimulated memory CD8^+^ T cells. Our results with DC-primed CD8^+^ T cells fit with a broad CTL response that could be required to clear latent HIV-1 in chronically infected individuals having a predominance of HIV-1 escape mutations (9). Alternatively, curing HIV-1 in such chronically infected persons on cART with a DC-based approach could require favoring CTL responses against low-entropy, conserved HIV-1 epitopes in Gag, which, as in acute HIV-1 infection, are superior to targeting high-entropy, variable regions that rapidly lead to CTL escape mutations (59, 60).

Our study supports the notion that induction of CTL responses from naive CD8^+^ T cell precursors, rather than a mere reactivation of memory CTL, will be required to effectively target the HIV-1 reservoir (24, 26). The study also highlights an important obstacle to cure HIV-1 infection in that the surviving antigenic epitope variants within the HIV-1 reservoir can act as partial T cell receptor agonists (61) to preferentially promote dysfunctional responses, i.e., partial activation of cross-reactive, memory CTL that display cytokine activity but do not kill target cells (24). This dysfunction can create barriers that could both hinder the induction of epitope-specific primary CTL responses to new variants and promote chronic inflammation that favors HIV-1 persistence. Therefore, the induction of effective CTL by DC could fall victim to “original antigenic sin,” in which existing, ineffective memory reactivity can obstruct the effective priming of new CTL against the relevant viral variant (62).

Cytokine secretion accompanied by varied or absent CTL killing is not a new phenomenon (63–65), nor is the expression of CD107a in the absence of IFN-γ production (66), highlighting the possibility that a single correlate of viral control may not exist and that an anti-HIV-1 CTL response is composed of a multitude of factors. Combined with findings from the present study, these observations suggest that existing memory CD8^+^ T cells that secrete proinflammatory factors in the absence of CTL killing could promote viral replication following antigen stimulation. This has important implications for DC-based immunotherapies that are to be implemented while participants are on cART and have memory T cells that are specific for variants of the immunotherapeutic antigen. While targeting the memory CD8^+^ T cell population can promote some antiviral cytotoxic effects (11), reactivation of ineffective memory T cell responders might actually contribute to viral spread and impede the generation of more-effective *de novo* CTL responses. It is imperative that these potential effects of a DC immunotherapy on the endogenous pool of HIV-1-specific memory CD8^+^ T cells be minimized. To this end, we propose that the type of DC used in our study, termed DC1 (34), has certain qualities that are superior to those that have been used with some success in HIV-1 therapeutic trials (33, 67) and could bias the immune system toward priming of *de novo* anti-HIV-1 CTL. DC1 are mature, have lymph node homing properties, and are specifically programmed to be superior in their: (i) responsiveness to the T cell signal CD40L, with production of IL-12p70 (30, 34); (ii) antigen-processing function (68–70); (iii) ability to selectively attract and promote interaction with naive T cells rather than regulatory T cells by, respectively, producing chemokine (C-C motif) ligand 19 (CCL19) and not CCL22 (71, 72); (iv) ability to “reticulate” and form intercellular networks (34, 73) required to facilitate effective exchange of antigenic information with lymph node-resident DC (74); and (v) high expression of programmed cell death 1 ligand 1 that can engage programmed death 1, which is not expressed on naive T cells but remains upregulated on memory CD8^+^ T cells during cART (75, 76), rendering these cells highly susceptible to apoptosis (77). Thus, DC1 are uniquely equipped to preferentially drive *de novo* Th1- and CTL-mediated immune responses (24, 30, 78).

The findings presented here suggest that the dysfunction of existing memory CTL during cART compared to the superior antiviral effects of *de novo*-generated CTL could represent a previously unappreciated challenge toward the development of effective HIV-1 immunotherapies. Our findings should be interpreted with caution, as they are limited to our *in vitro*-tested research participants and are not necessarily indicative of immunotherapeutic success *in vivo*. Moreover, it is important to consider the fact that these participants were chronically infected for a substantial period of time (ranging approximately 7 to 10 years) before initiating cART. It is conceivable that such a delay in therapy could greatly affect the functional quality of memory CTL and that early initiation of cART after HIV-1 infection could prevent extensive memory T cell escape and dysfunction. Nevertheless, our findings are encouraging and potentially paradigm-shifting, i.e., naive CD8^+^ T cells in chronically HIV-1-infected persons on long-term cART have the repertoire and function necessary to respond to a primary HIV-1-specific stimulation. This study serves as an initial proof of principle that naive CD8^+^ T cells in chronically infected persons on cART can successfully respond to a DC-based immunotherapy and supports further research evaluating the use of DC as a means to specifically target naive CD8^+^ T cells, while sparing reactivation of memory CD8^+^ T cells.

## MATERIALS AND METHODS

### Study participants.

Three HIV-1-infected participants, S2, S3 and S8, were chosen from the Multicenter AIDS Cohort Study (MACS), a natural history study of men who have sex with men (37, 38). Where indicated, anonymous healthy, HIV-1-negative, blood buffy coat donors were used in DC phenotyping experiments. Human subject research was approved by the University of Pittsburgh Institutional Review Board, and informed consent was obtained from all participants. The HIV-1-positive MACS participants were chosen on the basis of their prolonged, documented pre- and postinfection history in the study, typical course of disease progression prior to cART, and favorable response to cART (see Fig. S1 in the supplemental material) (12). HLA haplotypes were determined for each participant by the Tissue Typing Laboratory at the University of Pittsburgh Medical Center. Seropositivity was confirmed by positive enzyme-linked immunosorbent assay (ELISA) for the presence of HIV-1 p24 and a Western blot with bands corresponding to at least two of the Gag, Pol, and Env proteins (38). Blood specimens and epidemiological and clinical data were collected at each visit, as described previously (12). All three participants progressed to AIDS as defined by the CDC (<200 CD4^+^ T cells/mm^3^) within 8.3 years after seroconversion. These participants received cART and maintained plasma HIV-1 RNA below 20 copies/ml at most post-cART visits.

### Isolation of monocytes and peripheral blood lymphocytes.

PBMC were obtained from healthy, HIV-1-negative buffy coat donors, or by leukapheresis of the three HIV-1-infected participants. PBMC were isolated using Ficoll Hypaque density separation and were further separated into monocytes and peripheral blood lymphocytes (PBL) by Percoll density separation. Monocytes and PBL were frozen in fetal bovine serum (FBS) with 10% dimethyl sulfoxide (DMSO) in aliquots of 10 × 10^6^ cells/vial. Fresh PBL were used immediately for purification of naive and memory CD4^+^ and CD8^+^ T cells.

### Induction and isolation of the autologous HIV-1 from latently infected CD4^+^ T cells.

We used a previously described virus culture assay to induce HIV-1 production by latently infected CD4^+^ T cells obtained during or immediately prior to cART (79, 80) in participants S2, S3, and S8. The presence of HIV-1 in culture supernatants was evaluated every 3 days by p24 ELISA (Zeptometrix, Buffalo, NY). Cultures were terminated and supernatants were collected when the concentration of p24 reached or exceeded 20,000 pg/ml. The virus was passed five times through centrifugal filtration devices (Millipore, Billerica, MA) to remove any contaminating cytokines from the prolonged cell culture. The purified virus was then resuspended in RPMI 1640 medium supplemented with 10% FBS and frozen at −80°C. A p24 ELISA was performed on each aliquot to determine the concentration of purified virus in each sample.

### HIV-1 sequencing and peptide synthesis.

Autologous HIV-1 *gag* sequencing was performed on virus isolates from all three participants after long-term cART. For this, viral RNA was manually extracted from cell culture supernatants using a viral RNA minikit (Qiagen, Valencia, CA). cDNA synthesis was performed using Nef3 (81) and RT2 (82) primers with SuperScript III reverse transcriptase (200 U/ml; Invitrogen, Carlsbad, CA). Endpoint dilution of cDNA was used prior to viral gene amplification to avoid template resampling and detection of PCR-derived errors upon sequencing (83, 84). First-round PCR was performed with the Gag1 (82) and RT2 primers in a multiplex reaction. Singleplex second-round PCR was performed with Gag2 (82) and RSP15R (85) primers. PCR products were examined on a QIAxcel automated electrophoresis system (Qiagen), and Sanger sequencing was performed on samples with positive bands (High Throughput Genomics Center, Seattle, WA).

A library of 18-mers representing the consensus autologous Gag sequence detected during cART was generated using PeptGen on the Los Alamos HIV Molecular Immunology Database (LANL DB) website (http://www.hiv.lanl.gov/content/immunology) and synthesized for each participant (Sigma-Aldrich, St. Louis, MO). Peptides were resuspended in 50 µl sterile DMSO and were frozen in AIM V medium at 1 mg/ml at −80°C.

### Calculation of Shannon entropy scores.

For each 18-mer peptide, an overall entropy score was computed as the median of the site entropies (http://www.hiv.lanl.gov/content/sequence/ENTROPY/entropy_readme.html) for each position in the LANL HIV Sequence Database 2014 Gag protein “filtered web alignment” (http://www.hiv.lanl.gov/content/sequence/NEWALIGN/align.html) that was overlapped by the peptide. The original alignment contained 3,638 sequences and 651 columns; we report here entropies calculated from the B clade sequences only (1,117 sequences).

### Purification of naive and memory CD4^+^ and CD8^+^ T cells.

Fresh PBL were resuspended in phosphate-buffered saline (PBS) and stained with CD3 peridinin chlorophyll protein (PerCP) (BD Pharmingen, San Jose, CA), CD4 Pacific Blue (BD Pharmingen), CD8 PerCp-Cy5.5 (BD Pharmingen), CD45RA allophycocyanin (APC)-Cy7 (BD Pharmingen), CD62L APC (BD Pharmingen), CD31 phycoerythrin (PE) (BD Pharmingen), and CCR7 fluorescein isothiocyanate (FITC) (R&D Systems, Minneapolis, MN). Cells were washed with PBS and resuspended at 10^7^/ml in Iscove’s modified Dulbecco’s medium (IMDM) containing 10% FBS (IMDM−10% FBS). Naive CD4^+^ (CD3^+^, CD4^+^, CD45RA^+^, CD62L^+^, CCR7^+^, or CD31^+^) and naive CD8^+^ (CD3^+^, CD8^+^, CD45RA^+^, CD62L^+^, or CCR7^+^) T cells, as well as the nonnaive CD4^+^ and CD8^+^ T cells were purified to >97% using a BD FACSAria IIU cell sorter (see Fig. S2A in the supplemental material). Purified naive populations were tested for the presence of contaminating memory cells in an overnight IFN-γ ELISpot assay using a combination of cytomegalovirus, Epstein-Barr virus, and influenza A virus peptides (CEF) as antigens (Fig. S2B).

In a second set of experiments, stem cell memory T (Tscm) CD8^+^ and CD4^+^ T cells were removed from the naive population using the Tscm marker CD95, according to the following staining and gating strategy: frozen PBL were thawed and stained with CD3 APC-H7, CD4 V450, CD8 PerCP-Cy5.5, CD45RA PE-Cy7, CCR7 PE (R&D Systems, Minneapolis, MN), CD27 APC, and CD95 FITC (all BD Pharmingen, San Jose, CA). Cells were washed with PBS and resuspended at 10^7^/ml in IMDM−10% FBS. Naive CD4^+^ (CD3^+^, CD4^+^, CD45RA^+^, CD27^+^, CCR7^+^, or CD95_¯_) and naive CD8^+^ (CD3^+^, CD8^+^, CD45RA^+^, CD27^+^, CCR7^+^, or CD95_¯_) T cells, as well as the nonnaive CD4^+^ and CD8^+^ T cells were purified to >97% using a BD FACSAria IIU cell sorter (see Fig. S2C in the supplemental material). These naive populations were also tested for the residual presence of contaminating memory cells by measuring CD107a surface expression by CD8^+^ T cells after overnight stimulation in the presence of CEF mix of peptides or a mix of overlapping peptides covering autologous HIV Gag sequences (Fig. S2D).

### Generation of monocyte-derived DC.

Monocyte-derived DC were generated from HIV-1-negative donors and each HIV-1-infected participant as previously described (26). For priming and/or DC stimulation using whole virus, ~0.5 million immature DC were incubated with 50 ng of purified autologous aldrithiol-2 (AT-2)-inactivated HIV-1 for 2 h (40). Immature DC were treated with recombinant CD40L (0.5 µg/ml; Enzo, Farmingdale, NY) and IFN-γ (1,000 U/ml; R&D Systems, Minneapolis, MN) for 48 h. Mature, antigen-loaded DC were harvested and gamma irradiated (3,000 rads) to kill any contaminating T cells in the DC cultures.

### DC phenotyping.

The maturation status of DC generated from HIV-1-negative donors was evaluated by flow cytometry staining for surface expression of CD86, CD83, CD40, OX40L, and CCR7 (BD Pharmingen). The responsiveness of mature DC to subsequent CD40L stimulation was evaluated by adding soluble CD40L (0.5 µg/ml) or media to mature DC and incubating for 24 h. Supernatants were harvested and evaluated by IL-12p70 ELISA as previously described (24).

### Generation of DC-primed naive and DC-stimulated memory CTL effector populations.

Mature, antigen-loaded DC were cocultured with T cells isolated following either of the two sorting strategies outlined in Fig. S2 in the supplemental material. Thus, naive CD4^+^ and CD8^+^ T cells obtained post-cART to generate “DC-primed naive” T cells and memory CD4^+^ and CD8^+^ T cells obtained post-cART to generate “DC-stimulated memory” T cells at a dC:T (ratio of DC-primed naive T cell to DC-stimulated memory T cells) cell ratio of 1:10. DC priming of naive and stimulation of memory T cells in HIV-1-infected participants on cART was performed with the addition of soluble CD40L (0.5 µg/ml) at the time of coculture of DC-primed naive T cells and DC-stimulated memory T cells. The cultures were allowed to grow for 12 days. The cultures were supplemented with recombinant IL-2 (100 IU/ml; Chiron, Emeryville, CA), IL-7 (10 ng/ml; Miltenyi, Auburn, CA), and IL-15 (2.5 ng/ml; PeproTech, Rocky Hill, NJ) on day 3 and every 3 days thereafter, and the cells in culture were split as needed with IMDM−10% FBS supplemented with 10 µM efavirenz (EFV). EFV was obtained through the NIH AIDS Reagent Program, Division of AIDS, NIAID, NIH. T cell cultures were then restimulated with mature, autologous DC loaded with the viral antigen that was used in the initial stimulation. These cultures were grown for an additional 7 days, again supplementing with IL-2, IL-7, and IL-15 every 3 days and splitting with media supplemented with EFV. The efficacy of EFV and the absence of reactivated endogenous HIV-1 in the supernatant were confirmed by p24 ELISA. *Ex vivo* T cells were generated by isolating bulk T cells from contemporaneous PBMC using a negative T cell enrichment kit (EasySep; STEMCELL Technologies) and culturing in IMDM−10% FBS supplemented with IL-2 (100 IU/ml) and IL-7 (10 ng/ml) for 2 days. This “*ex vivo*” condition served as a baseline control for the endogenous HIV-1-specific T cell response. *Ex vivo* T cells and DC-stimulated primary and memory T cells were generated in triplicate and used in functional assays.

### Generation of autologous HIV-1-infected CD4^+^ T cells.

Autologous CD4^+^ T cells were superinfected with autologous HIV-1 as described previously with minor modifications (48, 86). Contemporaneous PBMC were depleted of CD8^+^ T cells using a CD8 T cell positive isolation kit (EasySep; STEMCELL Technologies). The CD8-negative (CD8^neg)^ population was cultured for 2 days in IMDM−10% FBS in the presence of IL-2 (100 IU/ml) and phytohemagglutinin (PHA) (1 µg/ml; Sigma-Aldrich) to induce T cell activation. Activated cells were washed and incubated for 1 h in IMDM−10% FBS containing Polybrene (5 µg/ml). Cells were again washed and resuspended in the concentration of purified autologous HIV-1 that resulted in 10 to 30% of the cells being infected after an additional 3 days in IMDM−10% FBS and IL-2 (100 IU/ml). After 3 days of incubation, CD4^+^ T cells were isolated by negative selection using a CD4^+^ T cell enrichment kit (EasySep; STEMCELL Technologies) and were stained for surface expression of CD8-PerCP-Cy5.5 (BD Pharmingen) and intracellular expression of HIV-1 core antigens using the KC57-FITC (KC57 labeled with FITC) antibody (Beckman Coulter, Brea, CA) per the manufacturer’s instructions to confirm we had generated a pure population of CD8^neg^ T cells, of which 10 to 30% were positive for HIV-1.

### HIV-1-specific ELISpot and HIV-1-infected cell elimination assay.

CD8^+^ T cells were isolated from DC-primed naive and DC-stimulated memory cultures by negative selection using a custom CD8^+^ T cell enrichment kit without the CD56 marker (EasySep; STEMCELL Technologies) and were immediately evaluated for IFN-γ production and effector function. IFN-γ ELISpot assays were performed as previously described (21, 26) by stimulating with immature DC loaded with individual overlapping Gag 18-mers (1 µg/ml) at a stimulator/responder ratio of 1:10. All ELISpot assays included negative-control wells with T cells or T cells and immature DC without peptide. ELISpot data were calculated as the means of spots in duplicate wells minus the mean plus 2 standard deviations of spots in duplicate negative controls. CTL effector function was assessed by coculture of purified CD8^+^ T cells from DC-primed naive and DC-stimulated memory cultures with fresh, autologous, infected CD4^+^ T cells at various effector/target ratios for 18 h at 37°C. The baseline percentage of infection was determined by incubation of infected CD4^+^ T cells without CD8^+^ T cells. Cocultures were harvested and stained for surface expression of CD8-PerCP-Cy5.5 (BD Pharmingen) and intracellular expression of HIV-1 core antigens using the KC57-FITC antibody (Beckman Coulter) per the manufacturer’s instructions. Samples were assessed on a BD LSRFortessa flow cytometer (BD Biosciences) and analyzed using FlowJo version 9.6.4. The percentage of infected CD4^+^ T cells was determined by gating on the CD8^neg^ population and then on the KC57 (HIV core antigen)-positive subset (48). The percent reduction in infected CD4^+^ T cells was determined for each condition at each E:T ratio and is in relation to the baseline infection of targets alone. The percent reduction in infected targets at each E:T ratio was transformed to effector units 20% (EU_20_), i.e., lytic units calculated based on the relative number of effectors required to yield 20% killing of the target cells (49).

### HIV-1 peptide-specific killing assay.

Briefly, autologous CD4 cells were stained with either CFSE or CellTrace violet dyes (Invitrogen) following the manufacturer’s protocols. Target cells were then loaded (CFSE) or not loaded (violet) with individual peptides at 100 ng/ml in PBS for 60 min at room temperature (RT); excess unbound peptide was removed by three washes in PBS. Peptide-loaded targets were then incubated overnight at various E:T ratios (10:1, 3:1, and 1:1) with CTL derived from DC-primed naive or DC-stimulated memory cultures. After incubation, events were acquired with a BD LSRFortessa flow cytometer (BD Biosciences) and analyzed using FlowJo version 9.6.4. The percentage of killing at each E:T ratio was calculated based on the number of events gated for each of the two colored target populations using the following equation: percentage of specific killing = [1 − (number of CFSE events/number of violet events)] × 100.

### Computational analyses.

Known MHC class I-restricted HIV-1 Gag epitopes were identified using the Los Alamos CTL/CD8^+^ T Cell Epitope Database (87). Computational identification of predicted epitopes within 18-mers was performed using netMHCpan version 2.8 (88) for each HLA allele. Predicted epitopes were defined as peptides 8 to 11 amino acids in length that exhibited weak or strong predicted affinity for the cognate HLA allele as determined by the 50% inhibitory concentration (IC_50_).

### Statistical analyses.

Paired *t* tests were used to compare IFN-γ production by DC-primed naive and DC-stimulated memory T cells to autologous p17 and p24 peptide antigens. Effector units in DC-primed naive and DC-stimulated memory CD8^+^ T cells were compared using the Student’s *t* test. All figures and statistics were generated using GraphPad Prism 6 (GraphPad Software, Inc., La Jolla, CA), except the entropy figures and statistics, which were generated in R (47).

### Nucleotide sequence accession numbers.

HIV-1 Gag sequences were deposited in GenBank under accession numbers KX137177 to KX137215.

## SUPPLEMENTAL MATERIAL

Figure S1  CD4^+^ T cell counts and viral load history of the MACS participants. HIV-1 viral load is expressed as the number of RNA copies per milliliter. CD4^+^ T cell counts are expressed as absolute numbers/mm^3^. Study entry, initiation of therapy (cART), and time of leukapheresis are expressed in years and centered around the estimated time of seroconversion (i.e., midpoint between the last seronegative time point and first seropositive time point, 6-month interval, clinic visit) taken as the zero time value. Abbreviations: 3TC, lamivudine; 4dT, stavudine; ABC, abacavir: EFV, efavirenz; FTC, emtricitabine; IDV, indinavir; LPV, lopinavir; RTV, ritonavir; SQV, saquinavir; TDF, tenofovir; ZDV, zidovudine. Download Figure S1, TIF file, 0.2 MB

Figure S2  Gating strategy for sorting naive T cells. (A) Naive CD4^+^ (CD3^+^, CD4^+^, CD62L^+^, CD45RA^+^, CCR7^+^, and CD31^+^) and naive CD8^+^ (CD3^+^, CD8^+^, CD62L^+^, CD45RA^+^, and CCR7^+^) T cells were sorted from bulk PBL obtained from each participant during cART. Memory T cells were classified as those not falling within the naive CD4^+^ and CD8^+^ T cell gates. FSC, forward scatter. (B) Cells sorted as detailed above for panel A were stimulated overnight in the presence of peptide antigens CEF, and the number of IFN-γ-producing SFC per 10^6^ cells were determined by an ELIspot. (C) Gating strategy used to exclude Tscm (CD95^+^) for sorting naive CD4^+^ (CD3^+^, CD4^+^, CCR7^+^, CD45RA^+^, CD27^+^, and CD95^−^) and naive CD8^+^ (CD3^+^, CD4^+^, CCR7^+^, CD45RA^+^, CD27^+^, and CD95^−^) T cells from frozen PBL. Memory T cells were classified as those not falling within the naive CD4^+^ and CD8^+^ T cell gates. (D) Cells sorted as detailed above for panel C were stimulated overnight in the presence of peptide antigens CEF and HIV-1 Gag. The fraction of CD8^+^ T cells positive for CD107 is shown. Download Figure S2, TIF file, 0.2 MB

Figure S3  Comparison of entropy between targeted 18-mers within each study participant. (A) Mean amino acid (AA) site entropies of all tested peptides, categorized as shown in Fig. 4A and further subdivided by participant (S2, S3, and S8). (B) Entropy of reactive peptides. The differences between primary and memory responses per participant (sorted by entropy) are shown. For each 18-mer peptide tested, the mean site entropy was calculated for each column in the LANL HIV Sequence Database 2014 Gag protein “filtered web alignment” (http://www.hiv.lanl.gov/content/sequence/NEWALIGN/align.html) that was overlapped by the peptide. The original alignment contained 3,638 sequences and 651 columns; we report here entropies calculated from the B clade sequences only, i.e., 1,117 sequences. The entropy of each tested peptide is shown as a vertical bar, sorted left to right by the entropy score, and colored by the arithmetic difference between the DC-primed naive T cell response and the DC-stimulated memory T cell response (red shows higher-magnitude DC-primed naive T cell response, and blue shows higher-magnitude DC-stimulated memory T cell response; scale bars at right). Download Figure S3, TIF file, 0.2 MB

Figure S4  Peptide-based flow cytometry cytotoxicity assay. A flow cytometric cytotoxicity assay was developed where CD8^+^ T cell targets were differentially labeled with dyes violet 450 (violet) and CFSE (green). The CFSE-labeled cells were loaded with the peptide of interest, while the violet 450-labeled cells served as controls. CTL killing activity was determined after coincubation by measuring specific loss of the CFSE-labeled cells by flow cytometry, and results were converted into effector units (EU). Download Figure S4, TIF file, 0.1 MB
