# Erratum for Smith et al., Effective Cytotoxic T Lymphocyte Targeting of Persistent HIV-1 during Antiretroviral Therapy Requires Priming of Naive CD8^+^ T Cells

**DOI:** 10.1128/mBio.01012-16

**Published:** 2016-07-12

**Authors:** Kellie N. Smith, Robbie B. Mailliard, Paolo A. Piazza, Will Fischer, Bette T. Korber, Ronald J. Fecek, Deena Ratner, Phalguni Gupta, James I. Mullins, Charles R. Rinaldo

**Affiliations:** a Department of Microbiology and Molecular Genetics, University of Pittsburgh, Pittsburgh, Pennsylvania, USA; b Department of Infectious Diseases and Microbiology, University of Pittsburgh, Pittsburgh, Pennsylvania, USA; c Theoretical Biology and Biophysics Group, Los Alamos National Laboratory, Los Alamos, New Mexico, USA; d Department of Microbiology, University of Washington, Seattle, Washington, USA; e Department of Pathology, University of Pittsburgh School of Medicine, Pittsburgh, Pennsylvania, USA

## ERRATUM

Volume 7, no. 3, doi:10.1128/mBio.00473-16, 2016. For transparency, the legend to Fig. 7 has been revised to include additional information that was inadvertently omitted. Below is the corrected version.

**FIG 7 ** DC-primed naive compared to DC-stimulated memory CD8^+^ T cells show higher CTL activity against targets loaded with select 18-mers from the autologous cART Gag p17/p24 sequences. (A) DC-primed naive and DC-stimulated memory CD8^+^ T cells were assessed by flow cytometry for cytolytic activity against autologous CD4^+^ T cells loaded with peptides found to be associated with a range of magnitudes of IFN-γ ELISpot responses (see Tables 1 to 3). The numbers of spots are reported within the parentheses below each peptide sequence for DC-stimulated memory T cells (before the slash) and DC-primed naïve T cells (after the slash). Shannon entropy scores are in parentheses to the right of each peptide. The bottom data set within the vertical panels (peptides EV18, QS18, RL18) shows CTL activity against peptides that were not associated with either DC-stimulated memory or DC-primed naive IFN-γ production. For each peptide, the relevant epitope sequence is shown in red, and the HLA-restricting allele is shown below each sequence. Data are means plus standard errors of 3 or 4 replicates within the CTL assay of DC-stimulated and DC-primed naive CD8^+^ T cells (*, *P* < 0.05; **, *P* < 0.01). (B) (Left) Flow cytometry analysis of CD107a and CD8 surface expression levels of DC-stimulated memory CD8^+^ T cells from participant S2 following exposure to autologous HIV-1 founder epitope peptide (LFNTVATLY) and a reservoir-associated variant (LYNTIATLY [mutant amino acids underlined]). (Right) The bar graph represents relative decrease in CD8 expression (triplicates; mean fluorescence intensity [MFI] ± SD) between antigen-responsive CD8^+^ T cells before and after antigen stimulation (*P* < 0.05). (C) Cytotoxicity of autologous CD4^+^ T cell targets expressing either the HIV-1 founder epitope LFNTVATLY or reservoir variant epitope LYNTIATLY following 18-h incubation with S2 DC-stimulated memory CD8^+^ T cells (triplicates; mean ± SD; *P* < 0.05).

