# Supplementary figures and images for: Effective Cytotoxic T Lymphocyte Targeting of Persistent HIV-1 during Antiretroviral Therapy Requires Priming of Naive CD8+ T Cells
Source: mBio. 2016 May 31;7(3):e00473-16. doi: 10.1128/mBio.00473-16 (PMC4895106; doi:10.1128/mBio.00473-16)

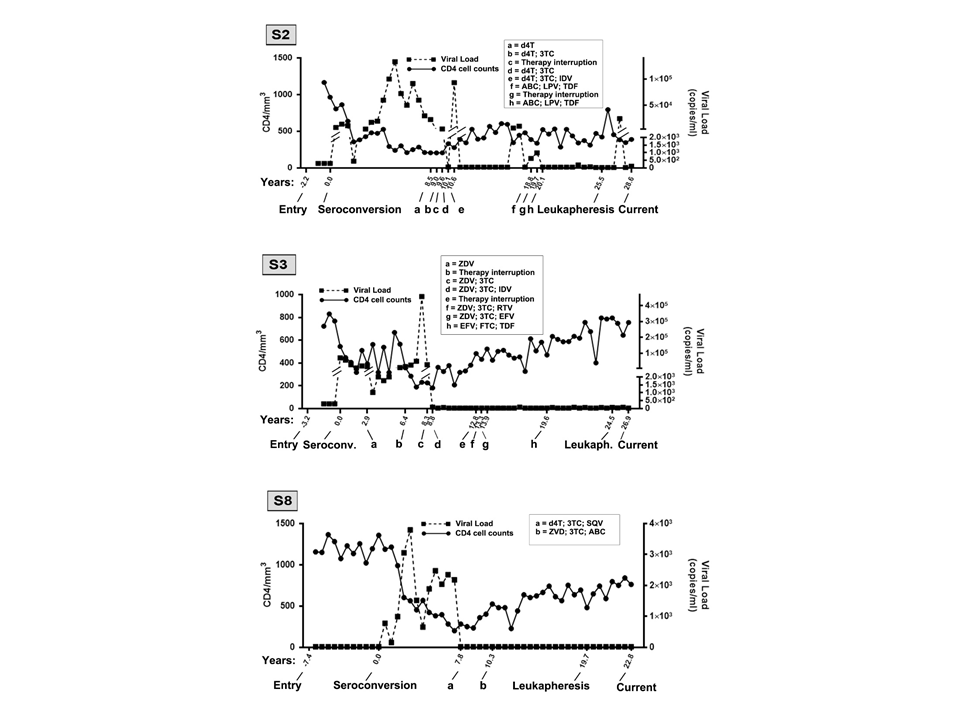

Supplement: Figure S1 — CD4+ T cell counts and viral load history of the MACS participants. HIV-1 viral load is expressed as the number of RNA copies per milliliter. CD4+ T cell counts are expressed as absolute numbers/mm3. Study entry, initiation of therapy (cART), and time of leukapheresis are expressed in years and centered around the estimated time of seroconversion (i.e., midpoint between the last seronegative time point and first seropositive time point, 6-month interval, clinic visit) taken as the zero time value. Abbreviations: 3TC, lamivudine; 4dT, stavudine; ABC, abacavir: EFV, efavirenz; FTC, emtricitabine; IDV, indinavir; LPV, lopinavir; RTV, ritonavir; SQV, saquinavir; TDF, tenofovir; ZDV, zidovudine. Download [file mbo003162833sf1.tif]

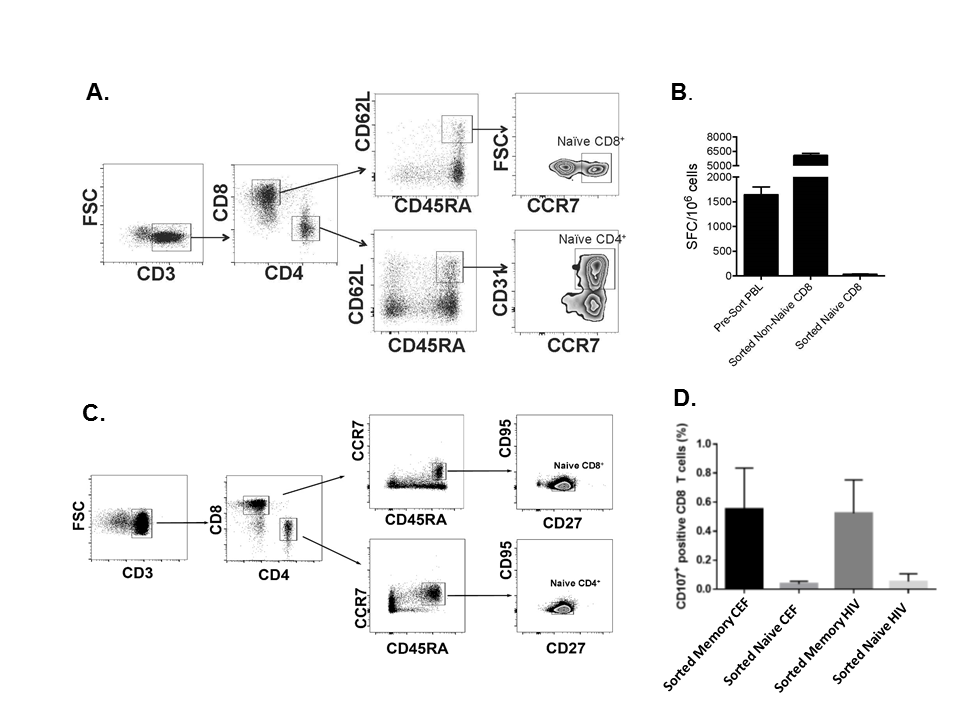

Supplement: Figure S2 — Gating strategy for sorting naive T cells. (A) Naive CD4+ (CD3+, CD4+, CD62L+, CD45RA+, CCR7+, and CD31+) and naive CD8+ (CD3+, CD8+, CD62L+, CD45RA+, and CCR7+) T cells were sorted from bulk PBL obtained from each participant during cART. Memory T cells were classified as those not falling within the naive CD4+ and CD8+ T cell gates. FSC, forward scatter. (B) Cells sorted as detailed above for panel A were stimulated overnight in the presence of peptide antigens CEF, and the number of IFN-γ-producing SFC per 106 cells were determined by an ELIspot. (C) Gating strategy used to exclude Tscm (CD95+) for sorting naive CD4+ (CD3+, CD4+, CCR7+, CD45RA+, CD27+, and CD95−) and naive CD8+ (CD3+, CD4+, CCR7+, CD45RA+, CD27+, and CD95−) T cells from frozen PBL. Memory T cells were classified as those not falling within the naive CD4+ and CD8+ T cell gates. (D) Cells sorted as detailed above for panel C were stimulated overnight in the presence of peptide antigens CEF and HIV-1 Gag. The fraction of CD8+ T cells positive for CD107 is shown. Download [file mbo003162833sf2.tif]

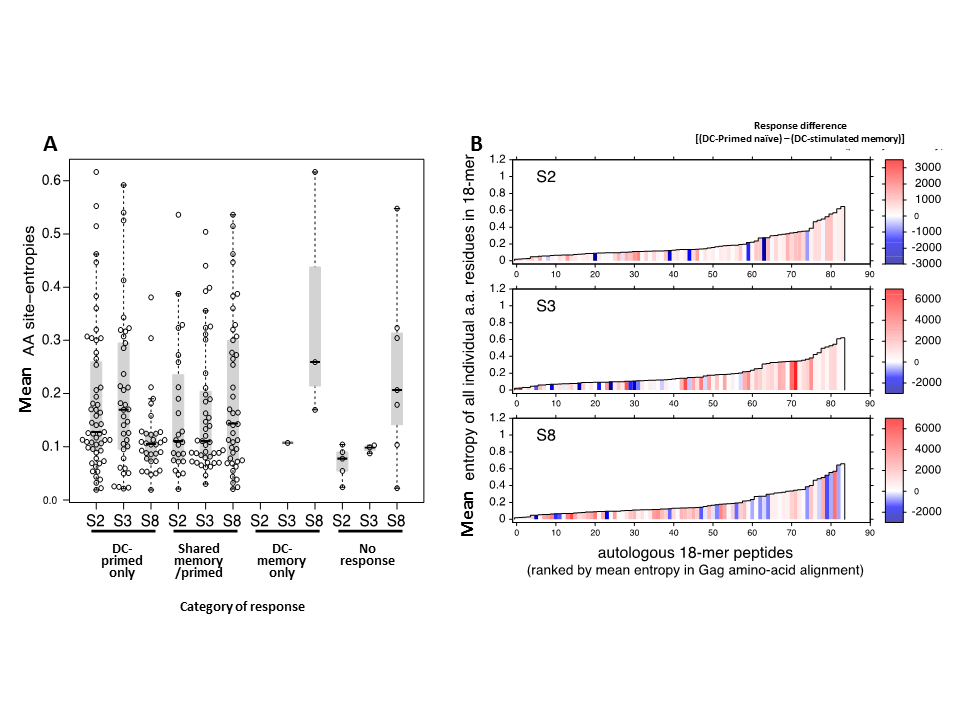

Supplement: Figure S3 — Comparison of entropy between targeted 18-mers within each study participant. (A) Mean amino acid (AA) site entropies of all tested peptides, categorized as shown in Fig. 4A and further subdivided by participant (S2, S3, and S8). (B) Entropy of reactive peptides. The differences between primary and memory responses per participant (sorted by entropy) are shown. For each 18-mer peptide tested, the mean site entropy was calculated for each column in the LANL HIV Sequence Database 2014 Gag protein “filtered web alignment” (http://www.hiv.lanl.gov/content/sequence/NEWALIGN/align.html) that was overlapped by the peptide. The original alignment contained 3,638 sequences and 651 columns; we report here entropies calculated from the B clade sequences only, i.e., 1,117 sequences. The entropy of each tested peptide is shown as a vertical bar, sorted left to right by the entropy score, and colored by the arithmetic difference between the DC-primed naive T cell response and the DC-stimulated memory T cell response (red shows higher-magnitude DC-primed naive T cell response, and blue shows higher-magnitude DC-stimulated memory T cell response; scale bars at right). Download [file mbo003162833sf3.tif]

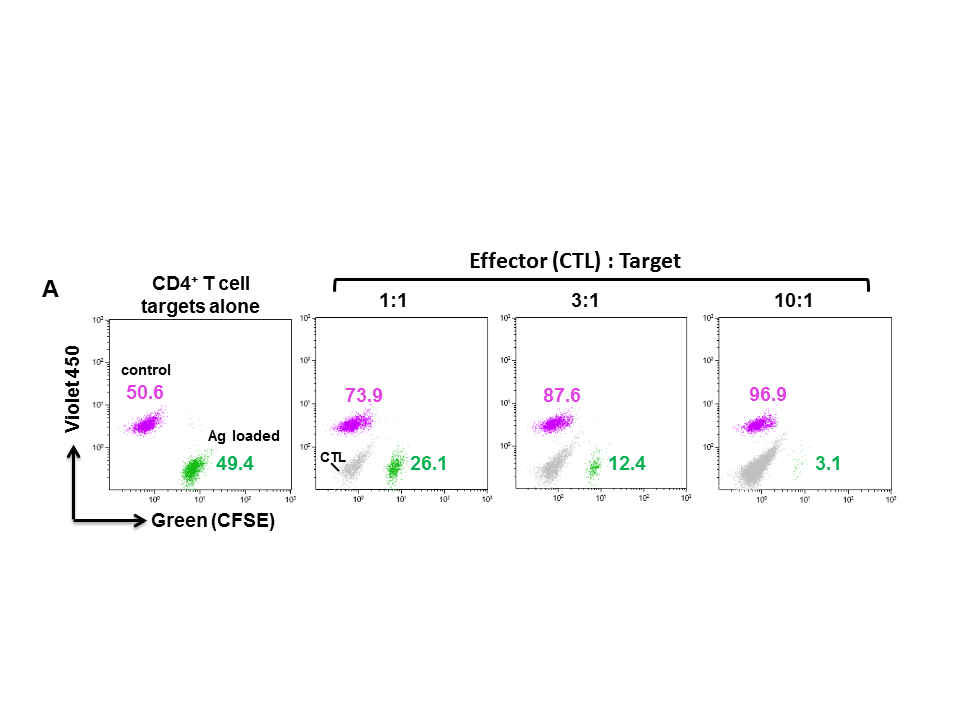

Supplement: Figure S4 — Peptide-based flow cytometry cytotoxicity assay. A flow cytometric cytotoxicity assay was developed where CD8+ T cell targets were differentially labeled with dyes violet 450 (violet) and CFSE (green). The CFSE-labeled cells were loaded with the peptide of interest, while the violet 450-labeled cells served as controls. CTL killing activity was determined after coincubation by measuring specific loss of the CFSE-labeled cells by flow cytometry, and results were converted into effector units (EU). Download [file mbo003162833sf4.tif]
